# Malignancy risk in Australian rheumatoid arthritis patients treated with anti-tumour necrosis factor therapy: analysis of the Australian Rheumatology Association Database (ARAD) prospective cohort study

**DOI:** 10.1186/s12891-015-0772-2

**Published:** 2015-10-20

**Authors:** Rachelle Buchbinder, Sharon Van Doornum, Margaret Staples, Marissa Lassere, Lyn March

**Affiliations:** Department of Epidemiology and Preventive Medicine, School of Public Health and Preventive Medicine, Monash University, Melbourne, Australia; Monash Department of Clinical Epidemiology, Cabrini Institute, Melbourne, Australia; Department of Medicine (Royal Melbourne Hospital), The University of Melbourne, Melbourne, Australia; St George Hospital, University of New South Wales, Sydney, Australia; Florance and Cope Professorial Department of Rheumatology, Royal North Shore Hospital, Institute of Bone and Joint Research, University of Sydney , Sydney, Australia

**Keywords:** Rheumatoid arthritis, Malignancy, Tumour necrosis factor, Biologic therapy

## Abstract

**Background:**

Malignancy risk with tumour necrosis factor inhibitor (TNFi) therapy remains unclear. Our primary aim was to assess malignancy risk with TNFi therapy in a cohort of Australian patients with rheumatoid arthritis (RA). We also assessed risk in a biologic-naïve group.

**Methods:**

Demographic data of all RA patients enrolled in the Australian Rheumatology Association Database before 25 October 2010 were matched to national cancer records in July 2010 (linkage complete to 2007). Verified self-reported malignancies occurring between 1 January 2008 and 25 October 2010 were also included in the analysis. Standardised incidence ratios (SIRs) were used to compare malignancy incidence in biologic-naïve and TNFi-exposed ARAD participants to the general population using site-, age- and sex-specific rates by calendar year. Rate ratios (RRs) were used to compare malignancy incidence in TNFi-exposed participants to biologic-naïve RA patients, and a composite RA cohort that included pre-TNFi person years, both adjusted for age, gender, smoking, methotrexate use and prior malignancy.

**Results:**

Forty-four malignancies were reported after 5752 person-years in the TNFi-exposed group (*N* = 2145) and 32 malignancies were reported after 1682 person-years in the biologic-naïve group (*N* = 803). No overall increased risk of malignancy in TNFi-treated RA patients was found when compared with the general population or with biologic-naïve RA patients. Compared to the biologic naïve group, without the inclusion of pre-TNFi years in the comparator group, the relative risk of female breast cancer was reduced in TNFi-treated patients (RR 0.17 (95 % CI 0.03 to 0.95)). It was no longer significant when adding pre-TNFi years in the comparator group. The risk of melanoma was increased for both biologic naïve and TNFi-treated patients when compared with the general population (SIR 2.72 (95 % CI 1.13 to 6.53) and SIR 2.03 (95 % CI 1.09 to 3.78) respectively). The relative risk of melanoma was not increased in the TNFi-exposed group compared with biologic naïve patients (RR 0.54, 95 % CI 0.12, 2.40). Inclusion of pre-TNFi person years in the comparator group did not change these results.

**Conclusions:**

Malignancy incidence was low in this RA cohort and biologic exposure did not increase the risk of malignancy. Melanoma risk was increased in both TNFi-treated and biologic-naïve RA patients compared with the general population suggesting that RA status, and possibly methotrexate exposure, may be responsible.

## Background

The issue of malignancy risk with tumour necrosis factor inhibitor (TNFi) therapy is complex. Chronic inflammation may promote tumorigenesis [1], and TNF has been demonstrated to both promote and inhibit tumour development in animal models depending on dose and biological conditions [2]. A systematic review of adalimumab and infliximab randomised controlled trials in rheumatoid arthritis (RA) patients reported a significantly higher malignancy risk compared with placebo [3]. Subsequent systematic reviews, which have included etanercept, certolizumab and golimumab trials, have shown no increased risk of invasive cancer [4–9].

While several groups have demonstrated an increased risk of lymphoma with TNFi therapy [10–12], which may just reflect the known increased risk in RA [13, 14], observational studies have generally reported no increased risk of malignancy overall [10, 11, 15–20]. A 2011 systematic review of prospective observational studies supported these findings (pooled estimate of overall risk of malignancy 0.95, 95 % CI 0.85-1.05 compared with RA TNFi-naïve controls), although the risk of non-melanoma skin cancer (NMSC) was increased (pooled estimate of risk 1.45, 95 % CI 1.15-1.76) [21]. A more recent systematic review examining the safety of biologic therapy included 49 prospective observational studies but was unable to pool data due to multiple sources of heterogeneity [22]. Based upon findings from individual studies they concluded that TNFi exposure is not associated with an increased overall risk of malignancy, lymphoma or NMSC but may be associated with a slight increased risk of melanoma based upon a single study [23].

In observational studies assessing risk of therapy the ideal comparator group should be identical to the study population in all ways apart from the intervention being evaluated. Outside the realms of randomised trials it is difficult to assemble such a group, and it is necessary to recognise limitations of different approaches. Prior investigators have compared the risk of malignancy in TNFi-treated RA patients with the general population [10, 11, 15–19], biologic-naïve RA cohorts [16, 17], and cohorts that include pre-TNFi person-years in the comparator group [10, 15]. Each of these comparison groups has potential differences compared to TNFi-treated RA patients, which may bias risk estimates. These include presence of rheumatic disease, disease activity, comorbidity and underlying malignancy risk. For instance, comparing malignancy risk in TNFi-treated RA patients with the general population measures not only the effect of TNFi therapy but also the effect of having RA. Comparing malignancy risk in TNFi-treated RA patients with biologic-naïve RA patients can partially account for the effect of RA, however confounding by indication can occur because TNFi-treated RA patients tend to have more severe disease than biologic-naïve RA patients. Including pre-TNFi person years (in individuals who subsequently commence TNFi therapy), can balance the groups in terms of disease severity, but separate biases may be introduced.

Because a history of malignancy can be a relative contra-indication for TNFi therapy, patients who are ultimately treated with a TNFi are likely to have a low incidence of malignancy in their pre-TNFi person years. This low incidence of malignancy in the comparator group can inflate the estimated malignancy risk in the TNFi-exposed group. Finally, while a number of biases and sample size issues could mask a true malignancy risk related to biologic therapy, alternative possibilities could be that there is no significant increased risk or that biologic therapy could be protective given the reduction in chronic inflammation and immune overactivity and the fact that TNF can both promote and prevent tumour formation through various complex mechanisms [24].

The aim of this study was to assess the risk of malignancy with TNFi therapy in a cohort of Australian RA patients. Australia has the highest risk of melanoma in the world [25], and a previous Australian study found a three-fold increased risk of melanoma in RA patients treated with methotrexate compared with the general population [26]. We therefore also assessed the risk of malignancy in a biologic-naïve group of RA patients to examine the relative contribution of the RA disease process and background RA treatments on malignancy risk.

## Methods

### Setting

In Australia, government-subsidised treatment with biological disease modifying anti-rheumatic drugs (bDMARDs) is subject to strict criteria. These criteria have changed over time, but include inadequate response to at least two traditional DMARDs (one of which must be methotrexate unless contraindicated) over a period of 6 months, a tender and swollen joint count of greater than 20 joints (or 4 large joints) and elevated inflammatory markers. These criteria limit bDMARD therapy to patients with highly active and treatment-resistant RA. Up until November 2007 TNFi therapy was the mandatory first choice of bDMARD unless absolute contra-indications were present.

### Australian Rheumatology Association Database (ARAD) design and data collection

The structure, governance and content of ARAD have been described previously [27–30]. Initiated in 2001, ARAD is a voluntary registry that collects longitudinal health outcomes data from Australian patients with inflammatory arthritis. Most participants enrol when they commence biologics. Enrolment is also encouraged for those not starting biologic drugs but active targeting of these patients has only occurred on an ad hoc basis. Based on residential postcode, demographic and clinical characteristics participants appear to be nationally representative, with 246 (79 %) rheumatologists from all states and territories having contributed patients [28].

ARAD has received ethical approval from committees and organisations across Australia. All participants provide written permission to be contacted by ARAD investigators and written informed consent to participate in the registry and the associated linkages.

At patient enrolment details of diagnosis, disease status data (ESR, CRP and joint count) and the bDMARD prescribed (if applicable) are obtained from the treating rheumatologist. All ARAD participants complete detailed entry and six-monthly follow-up questionnaires (paper-based or online). Data collected from the participants include: demographic details, disease duration and severity, self-reported past and current medical history including malignancies and other chronic conditions, use of anti-rheumatic drugs including the date commenced, smoking and alcohol history, Assessment of Quality of Life (AQoL) [31] and arthritis-specific disability assessed by the Health Assessment Questionnaire (HAQ) [32]. In the event of missing or ambiguous data or reporting of a significant event such as malignancy, ARAD staff contact the patient and the rheumatologist and/or other treating doctor to verify the data.

For the purpose of this study, ARAD participants were eligible if they had rheumatologist-diagnosed RA and had enrolled in ARAD prior to 25 October 2010 (the analysis cut-off date). Participants were divided into two mutually exclusive groups: participants who were biologic-naïve for the entire duration of observation, and participants who commenced a TNFi (etanercept, adalimumab or infliximab) as their first line biologic therapy. Our dataset predated subsidised prescription of certolizumab and golimumab in Australia. Patients who commenced a non-TNFi biologic as first line therapy (*n* = 5) were excluded. Baseline characteristics, medical history, HAQ and AQoL were extracted from the questionnaire completed at ARAD entry. Some questions relating to baseline descriptive variables and comorbidity were only added to the baseline questionnaire in January 2006 and so were unavailable for participants who completed their baseline questionnaire prior to this time.

### Ascertainment of malignancy

The National Cancer Statistics Clearing House (NCSCH) collates details of all malignancies occurring in Australia apart from NMSCs [33]. Notification of malignancy to the state-based cancer registries has been mandatory in Australia since 1982. The state-based registries send identified data to the NCSCH for aggregation at a national level. The International Classification of Diseases, 10th Revision (ICD-10) is used to code site of malignancy. The ARAD patient database was linked with the NCSCH in July 2010 to ascertain all invasive malignancies in ARAD participants. At the time of linkage the NCSCH was complete from 1982 to 2007 and therefore we were able to ascertain all malignancies that occurred in ARAD participants from the time of their enrolment. The date of diagnosis of malignancy as recorded in the NCSCH was used in the analysis. Self-reported malignancies occurring after 31 December 2007 and verified by the treating doctor or pathology report were also included in the analysis with the date of diagnosis as the date of the doctor verification or the histology report.

### Data analysis

Person-years for biologic-naïve patients were calculated from the date of enrolment in ARAD until death or analysis cut-off date (25 October 2010). Person years of exposure to TNFis began at either the start date of the therapy or enrolment in ARAD (whichever was later) and continued until death or analysis cut-off date. Person-years prior to the commencement of TNFi were calculated for the TNFi-exposed group from enrolment in ARAD to the start date of the first TNFi. Patients taking a non-TNFi as first line biologic were excluded.

Standardised incidence ratios (SIRs) were used to compare the incidence of malignancy in biologic naïve and the TNFi-exposed ARAD participants with incidence in the Australian general population using the site-, age- and sex-specific rates by calendar year. Malignancy incidence in TNFi-exposed participants was compared with that in biologic naive participants with rate ratios calculated using the Mantel-Cox method with significance assessed using the log-rank test of the respective ‘time to malignancy’ distributions adjusted for age, sex, calendar year, smoking status, prior malignancy and methotrexate use. All data were analysed using Stata 11 [34].

## Results

There were 2957 RA patients eligible for study inclusion; 803 were biologic-naïve and 2154 had received TNFi therapy (first-line therapy: etanercept (*n* = 1129, 54 %), adalimumab (*n* = 829, 38 %), infliximab (*n* = 150, 7 %)). The characteristics of the biologic naïve group at entry to ARAD and the TNFi-exposed group at commencement of therapy are shown in Table 1. Those starting TNFi as a first biologic therapy were younger, had more active disease as indicated by higher median joint count and CRP (but not ESR), greater prior use of DMARDs, including methotrexate and prednisolone, and higher disability and lower quality of life as indicated by higher HAQ and AQoL scores respectively. Prior malignancies were less commonly reported (3.3 % compared with 7.6 %).

**Table 1:** Demographic and clinical details of RA participants

	Biologic-naïve patients^(a)^	TNFi-treated patients^(b)^	*p*-value
	*n* = 803	*n* = 2154	
Female, n (%)	567 (70.6)	1582 (73.4)	0.12*
Age in yrs, mean (SD)	61.2 (12.3)	55.6 (125)	<0.001**
Rheumatoid factor positive^(c)^, n (%)	465/547 (85.0)	1073/1326 (80.9)	0.04*
No. of prior DMARDs, mean (SD)	2.5 (1.8)	4.0 (2.0)	<0.001**
Ever used methotrexate, n (%)	614 (76.5)	1851 (85.9)	<0.001*
Ever used prednisolone, n (%)	452 (56.3)	1899 (88.2)	<0.001*
HAQ score, mean (SD)	1.16 (0.79)	1.37 (0.73)	<0.001**
(range 0–3, 0 = no disability)			
AQoL score, mean (SD)	0.53 (0.27)	0.49 (0.24)	<0.001**
(range 0–1, 1 = full health)			
Joint count, median (range)	14.5 (0, 59) (n = 286)	24 (0, 72) (n = 906)	<0.001***
ESR mm/h, median (range)	26 (1, 129) (n = 263)	29 (0, 134) (n = 834)	0.07***
CRP mg/L (median, range)	8.5 (0.4, 161) (n = 264)	17 (0.1, 206) (n = 836)	<0.001***
Current smoker, n (%)	90/776 (11.6)	300/2111 (14.2)	0.07*
Years since RA diagnosis, mean (SD)	13.0 (11.3)	13.7 (10.5)	0.12**
Prior malignancies^(d)^, n (%)	61 (7.6 %)	72 (3.3 %)	<0.001*

^(a)^ data from time first entered into registry unless otherwise indicated; ^(b)^ data from time first commenced TNFi treatment; ^(c)^ data incomplete: n is shown in table; ^(d)^ verified malignancies (excluding NMSC and in-situ cancers) recorded prior to commencement of the study period. *HAQ* health assessment questionnaire, *AQoL* assessment of quality of life; *ESR* erythrocyte sedimentation rate, *CRP* C-reactive protein.* *p*-value from Chi-squared test; ** *p*-value from *t*-test; *** *p*-value from Wilcoxon Rank Sum test

Follow up was 5752 and 1682 person-years for TNFi-exposed and biologic-naïve patients respectively. Forty-four malignancies were reported in the TNFi-exposed group and 32 in the biologic-naïve group. The overall malignancy risk for biologic-naïve participants was comparable to that expected on the basis of population rates but, on examination of site specific cancers, these patients had an elevated risk of melanoma (Observed = 5, Expected = 1.84; SIR 2.72 (95 % CI 1.13 to 6.53)) and lung cancer (Observed = 6, Expected = 2.41; SIR 2.49 (95 % CI 1.12 to 5.53)) (Table 2). The overall malignancy risk among TNFi-treated RA patients was not elevated in comparison with the general population (Table 2), but there was an increased risk of melanoma (Observed = 10, Expected = 4.92; SIR 2.03 (95 % 1.09 to 3.78)) and a reduced risk of colorectal cancer (Observed = 1, Expected = 7.75; SIR 0.13 (95 % CI 0.02 to 0.92)) in these patients.

**Table 2:** Risk of cancer in biologic-naïve and TNFi-treated RA patients compared with the Australian general population, and relative risk in the TNFi treated patients compared to biologic naïve patients

	Risk of cancer in biologic-naïve RA patients compared with the general population			Risk of cancer in TNFi-treated patients compared with the general population			Relative risk (95 % CI) of cancer in TNFi-treated RA patients compared with biologic-naïve RA patients
Site	O	E	SIR (95 % CI)	O	E	SIR (95 % CI)	RR (95 % CI)^b^
All invasive cancers	32	23.38	1.37 (0.97, 1.94)	44	56.78	0.77 (0.58, 1.04)	0.65 (0.37, 1.17)
Melanoma	5	1.84	2.72 (1.13, 6.53)	10	4.92	2.03 (1.09, 3.78)	0.54 (0.12, 2.40)
Lung	6	2.41	2.49 (1.12, 5.53)	4	5.23	0.77 (0.29, 2.04)	0.24 (0.05, 1.17)
Lymphoid cancers^a^	1	1.49	0.67 (0.09, 4.76)	6	3.54	1.69 (0.76, 3.77)	4.01 (0.18, 88.19)
Colorectal	2	3.41	0.59 (0.15, 2.35)	1	7.75	0.13 (0.02, 0.92)	0.06 (0.001, 3.28)
Prostate	5	3.38	1.48 (0.62, 3.55)	7	7.71	0.91 (0.43, 1.90)	1.21 (0.33, 4.45)
Female breast	5	3.32	1.51 (0.63, 3.62)	4	10.29	0.39 (0.15, 1.04)	0.17 (0.03, 0.95)

*TNFi* tumour necrosis factor inhibitor, *RA* rheumatoid arthritis, *SIR* standardised incidence rate, *NHL* non-Hodgkin’s lymphoma; ^a^Lymphoid cancers includes all leukaemias and lymphomas, *RR* relative risk; ^b^adjusted for age, sex, calendar year, smoking status, methotrexate use and prior malignancy

When the TNFi-treated RA patients were compared with the biologic-naïve RA cohort there were no significant differences in malignancy risk overall or for specific cancer sites. (Table 2). No difference in risk of melanoma was found between groups (RR 0.54 (95 % CI 0.12 to 2.40)).

In the analysis comparing malignancy risk in patients unexposed to biologics and including the biologic-naïve group plus pre-TNFi person years of observation for those who later commenced TNFi treatment, there was no overall increased risk of malignancy compared with the Australian population but as with the previous comparison, the melanoma and lung cancers risks were elevated (Table 3). Adding the pre-TNFi person years of observation into the biologic-naïve comparator group did not appreciably alter the RRs for all invasive cancers or for specific sites when comparing malignancy risk to the TNFi-treated RA patients (Table 3).

**Table 3:** Risk of cancer in biologic-naïve RA patients (including TNFi treated patients prior to TNFi exposure) compared with the general population, with relative risk in the TNFi treated patients compared to biologic naïve patients

	Risk of cancer in biologic-naïve RA patients (including TNFi treated patients prior to TNFi exposure) compared with the general population			TNFi-treated patients compared with no previous exposure to biologics
Site	O	E	SIR (95 % CI)	RR (95 % CI) ^b^
All invasive cancers	33	24.86	1.33 (0.94, 1.87)	0.70 (0.39, 1.25)
Melanoma	5	1.97	2.54 (1.06, 6.11)	0.68 (0.16, 2.87)
Lung	6	2.55	2.35 (1.06, 5.24)	0.26 (0.05, 1.27)
Lymphoid cancers^a^	1	1.58	0.63 (0.09, 4.49)	4.03 (0.18, 89.28)
Colorectal	2	3.6	0.56 (0.14, 2.22)	0.19 (0.003, 13.94)
Prostate	5	3.61	1.39 (0.58, 3.33)	1.20 (0.33, 4.40)
Female breast	5	3.58	1.40 (0.58, 3.36)	0.20 (0.04, 1.06)

*TNFi* tumour necrosis factor inhibitor, *RA* rheumatoid arthritis, *SIR* standardised incidence rate, *NHL* non-Hodgkin’s lymphoma; ^a^Lymphoid cancers includes all leukaemias and lymphomas; *RR* relative risk; ^b^ adjusted for age, sex, calendar year, smoking status, methotrexate use and prior malignancy

## Discussion

The incidence of malignancy was low in this cohort of RA patients. We found no overall increased malignancy risk when TNFi-treated RA patients were compared with the general population or with biologic-naïve RA patients, a result that is consistent with numerous previously reported observational studies [10–12, 15–21]. While the risk of female breast cancer was not reduced in TNFi-exposed RA patients in comparison with the general population, we did observe a reduced relative risk of female breast cancer in TNFi-treated RA patients compared with RA patients never exposed to biologics without (but not with) inclusion of the pre-TNFi person years of observation of the TNFi-treated RA patients in the biologic-naïve comparison group.

A reduced risk of female breast cancer in RA in comparison with the general population has previously been demonstrated in a number of studies [18, 19, 35–37]. In keeping with our findings, a recent study that comprised biologic-naïve RA patients recruited to the British Society for Rheumatology Biologics Register from 2002 to 2009 found no difference in risk in comparison with the general population (SIR 1.07 (95 % CI 0.72 to 1.52) [38]. On the other hand, a reduced risk of breast cancer associated with TNFi exposure has been noted in a large observational study of RA patients that used a propensity score with relevant covariates and cohort trimming to improve the balance between DMARD cohorts [39]. They identified a reduced adjusted risk of breast cancer with TNFi exposure compared with methotrexate (Hazard Ratio 0.09 (95 % CI 0.02 to 0.39). In keeping with the results from our study, data from the British, German and Swedish registries have also suggested a reduced risk of breast cancer associated with TNFi exposure [18, 37, 40]. Mercer et al. noted a non-statistically significant reduction compared with biologic-naïve patients [40]; while both Askling et al. and Strangfeld et al. noted a reduced risk in both TNFi-naïve and TNFi-exposed RA patients compared with the general population [18, 37]. As noted by Mercer et al., it is biologically plausible that TNF inhibition may slow or prevent breast cancer progression [40], although a phase 2 study of etanercept did not find any objective disease response in people with metastatic breast cancer [41].

While we did not find an increased risk of melanoma as a result of TNFi exposure, we did find an increased risk of melanoma in comparison with the general population in both biologic-naïve and TNFi-exposed patients (with or without including pre-TNF exposure years of TNFi-treated patients). These findings are consistent with a previous study that reported an increased the risk of melanoma in RA patients treated with methotrexate in an Australian cohort study (SIR 3.0, 95 % CI 1.2, 6.2) [26]. The majority of patients in both the biologic naïve (80.2 %) and TNFi-exposed groups (96.9 %) were exposed to methotrexate in our study. Taken together, these observations suggest that RA status, and possibly methotrexate exposure related to RA status, may be responsible for this observed increased melanoma risk.

Several observational studies have evaluated the risk of melanoma in TNFi-treated RA patients and have reported conflicting results. In a comparison of RA patients from the National Data Bank with the US general population, Wolfe et al. reported an elevated risk of melanoma in the RA group overall (SIR 1.7, 95 % CI 1.3-2.3), and a non-significant elevated risk of melanoma in association with biologic therapy (OR 2.3, 95 % CI 0.9-5.4) [19]. In contrast, a nationwide population based prospective cohort study from Sweden revealed no increased risk of melanoma in biologic-naïve RA patients compared with the general population (Hazard Ratio (HR) 1.2, 95 % CI 0.9-1.5), whereas their comparison between TNFi-treated patients and the biologic naïve cohort suggested an association between TNFi treatment and invasive melanoma (HR 1.5, 95 % CI 1.0-2.2) [23].

The risk of melanoma in RA patients not taking biologic agents compared with the general population was recently reviewed by Perkins et al. [42]. Eleven studies were identified from Sweden (*n* = 3), the USA/Canada (*n* = 3), Denmark, Scotland, Australia, Taiwan and Spain. The SIRs for melanoma in these studies ranged from 0.7-3.8, with the highest risks in Australia and Spain (SIR 3.8). The pooled analysis gave rise to a SIR of 1.01 (95 % CI 0.93-1.10), suggesting no increased risk. A more recent UK study of malignancy in RA patients receiving non-biologic DMARD therapy compared with the general population reported a SIR of 2.05 (95 % CI 0.94-3.90) for melanoma [38]. Data regarding exposure to specific medications such as methotrexate, genetic background including skin colour and relative ultraviolet light exposure was not able to be considered in these analyses but could be important for melanoma risk in particular.

A reduced risk of colorectal cancer in RA patients has been consistently reported [36], postulated to be related to non-steroidal anti-inflammatory drug use [43]. However, in keeping with our data, some studies have not reported a reduced risk in biologic naïve RA patients compared with the general population [38]. We found a reduced risk of colorectal cancer in TNFi-treated patients in comparison to the general population but the risk was not reduced in comparison with biologic-naïve patients. This is compatible with some studies that have reported non-significant reductions in TNFi-treated patients compared with the general population [18, 37, 40]. However compared with biologic-naïve RA patients, Dreyer et al. reported an increased risk of colorectal cancer with TNFi exposure (Hazard ration 2.52 (95 % CI 1.11 to 11.15) [20]. Further investigation is required to clarify the true risk of colorectal cancer with TNFi treatment.

Our finding of an increased risk of lung cancer in biologic-naïve RA patients compared with the general population is consistent with previous studies (e.g. [16]) . However, similar to other registry studies [19, 40], we did not find an increased risk of lung cancer related to TNFi exposure.

The comparison between the TNFi-exposed cohort and the ‘biologic-naïve’ RA patients ensures that both groups have RA as an underlying disease state, although the TNFi-exposed group are likely to have more severe and/or active disease. The comparison between the TNFi-exposed cohort and the biologic-naïve cohort plus the pre-TNFi person-years of the TNFi-exposed RA patients is likely to have more balanced groups in terms of RA severity and increases the power of the analysis by increasing the person years in the comparator group. However inclusion of the pre-TNFi person-years of the TNFi-exposed RA patients did not appreciably change our results.

Other observational studies have used different comparator groups to investigate malignancy risk with TNFi therapy including the general population, a separate cohort, and a biologic-naïve cohort with and without pre-TNF exposure years. This may account for some of the observed differences between studies. Comparison with the general population fails to account for possible confounding due to factors related to the disease or its management. RA patients taking non-biologic DMARDs have been reported to have different rates of some cancers compared with the general population [36, 38], and therefore comparisons of TNFi treated patients with the general population must take this into consideration. Our finding in relation to melanoma risk is an example of this. Choosing a separate, biologic-naïve cohort also has potential for selection bias as these patients tend to have lower disease activity and different rates of concurrent and/or prior DMARD use than TNFi-treated patients. On the other hand, combining pre-TNFi years with a separate biologic-naïve cohort may also be prone to selection bias if patients are less likely to be prescribed TNFi if they have had a previous malignancy (irrespective of disease activity).

Although a history of malignancy was not an absolute contraindication to prescribing TNFi therapy in Australia when these drugs were first introduced, TNFi-treated patients were less likely to have had a prior malignancy (Table 1), suggesting clinicians may have been wary of prescribing TNFi to people with a prior history of malignancy. This concern appears to have diminished over time as evidence about the long-term safety of TNFi accumulates, although current recommendations continue to advise avoidance of TNFi in people with a history of malignancy within the prior five years [44].

Strengths of our study include national representation of patients from most rheumatologists, and over 7500 patient years of follow up. Linkage of the ARAD database with the NCSCH ensures virtual complete ascertainment of malignancies between 1982 and the end of 2007. Between 1 January 2008 and 25 October 2010 (analysis cut off) we relied upon patient-reported malignancies that were subsequently verified by the treating doctor and/or histology reports. Under-ascertainment of malignancies during this period is possible, however both groups of RA patients were subject to these conditions making systematic bias unlikely.

The study was limited by the fact that data for the entire Australian TNFi-treated RA population are unavailable, and thus we are unable to determine whether our TNFi-treated cohort is representative of Australian TNFi-treated patients in general. In addition, we acknowledge that our analyses may be compromised by immortal time bias whereby patients already diagnosed with cancer do not enrol in ARAD. We have no way of assessing or addressing this bias.

Our patient numbers were not sufficient to analyse data for each TNFi separately hence we have presented pooled data. As our dataset predated subsidised prescription of certolizumab and golimumab in Australia our results may or may not be generalisable to these anti-TNF therapies. Furthermore, we do not have sufficiently detailed information on concomitant medication use, including precise duration of therapy and dose, so cannot explore in detail the effect of methotrexate and other DMARDs on malignancy risk. Although the comparison with biologic-naïve RA patients goes some way towards removing RA status as a potential confounder, the biologic-naïve RA patients had lower levels of disease activity than the TNFi-treated patients. This difference in disease activity could bias the estimates of malignancy risk, however would be most likely to inflate the risk associated with TNFi therapy. Lastly surveillance bias, whereby regular review of RA patients by rheumatologists leads to increased melanoma detection, could have contributed to our finding of increased risk of melanoma in RA patients versus the general population.

Despite the large number of patient years of follow-up malignancy incidence is low in this cohort and the study perhaps lacks power. Larger numbers of patients followed for a longer time period may be necessary to be completely confident that a real increase in malignancy risk in our setting does not exist.

## Conclusions

Our study supports previous reports that have suggested treatment with TNFi is not associated with an overall increased risk of malignancy. It has also verified a previous Australian report of an increased risk of melanoma in RA patients treated with methotrexate compared with the general population. Regular monitoring of RA patients for development of skin cancer is recommended.

## References

[1] Grivennikov S, Greten F, Karin M (2010). Immunity, inflammation, and cancer. Cell.

[2] Bertazza L, Mocellin S (2010). The dual role of tumor necrosis factor (TNF) in cancer biology. Curr Med Chem.

[3] Bongartz T, Sutton AJ, Sweeting MJ, Buchan I, Matteson EL, Montori V (2006). Anti-TNF antibody therapy in rheumatoid arthritis and the risk of serious infections and malignancies. Systematic review and meta-analysis of rare harmful effects in randomized controlled trials. JAMA.

[4] Alonso-Ruiz A, Pijoan J, Ansuategui E, Urkaregi A, Calabozo M, Quintana A (2008). Tumor necrosis factor alpha drugs in rheumatoid arthritis: systematic review and metaanalysis of efficacy and safety. BMC Musculoskelet Disord.

[5] Askling J, Fahrbach K, Nordstrom B, Ross S, Schmid C, Symmons D (2011). Cancer risk with tumor necrosis factor alpha (TNF) inhibitors: meta-analysis of randomized controlled trials of adalimumab, etanercept, and infliximab using patient level data. Pharmacoepidemiol Drug Saf.

[6] Leombruno J, Einarson T, Keystone E (2009). The safety of anti-tumour necrosis factor treatments in rheumatoid arthritis: meta and exposure-adjusted pooled analyses of serious adverse events. Ann Rheum Dis.

[7] Thompson A, Rieder S, Pope J (2011). Tumor necrosis factor therapy and the risk of serious infection and malignancy in patients with early rheumatoid arthritis: a meta-analysis of randomized controlled trials. Arthritis Rheum.

[8] Lopez-Olivo M, Tayar J, Martinez-Lopez J, Pollono E, Cueto J, Gonzales-Crespo M, et al. Risk of malignancies in patients with rheumatoid arthritis treated with biologic therapy: a meta-analysis. JAMA. 2012;308:898–908. 10.1001/2012.jama.10857 22948700

[9] Michaud T, Rho Y, Shamliyan T, Kuntz K, Choi H (2014). The comparative safety of Tumor Necrosis Factor Inhibitors in rheumatoid arthritis: A meta-analysis update of 44 trials. Am J Med.

[10] Geborek P, Bladstrom A, Turesson C, Gulfe A, Petersson I, Saxne T, et al. Tumour necrosis factor blockers do not increase overall tumour risk in patients with rheumatoid arthritis, but may be associated with an increased risk of lymphomas. Ann Rheum Dis. 2005;64:699–703. 10.1136/ard.2004.030528 PMC1755491 15695534

[11] Pallavicini F, Caporali R, Sarzi-Puttini P (2010). Tumour necrosis factor antagonist therapy and cancer development: analysis of the LORHEN registry. Autoimmun Rev.

[12] Askling J, Baecklund E, Granath F, Geborek P, Fored M, Backlin C, et al. Anti-tumour necrosis factor therapy in rheumatoid arthritis and risk of malignant lymphomas: relative risks and time trends in the Swedish Biologics Register. Ann Rheum Dis. 2009;68:648–53. 10.1136/ard.2007.085852 18467516

[13] Baecklund E, Askling J, Rosenquist R, Ekbom A, Klareskog L. Rheumatoid arthritis and malignant lymphomas. Curr Opin Rheumatol. 2004;16:254–61. 10.1097/00002281-200405000-00014 15103253

[14] Kaiser R (2008). Incidence of lymphoma in patients with rheumatoid arthritis: a systematic review of the literature. Clin Lymphoma Myeloma.

[15] Askling J, van Vollenhoven R, Granath F, Raaschou P, Fored C, Baecklund E, et al. Cancer risk in patients with rheumatoid arthritis treated with anti-tumor necrosis factor alpha therapies: does the risk change with the time since start of treatment? Arthritis Rheum. 2009;60:3180–9. 10.1002/art.24941 19877027

[16] Carmona L, Abasolo L, Descalzo M, Pérez-Zafrilla B, Sellas A, de Abajo F, et al. Cancer in patients with rheumatic diseases exposed to TNF antagonists. Sem Arthritis Rheum. 2011;41:71–80. 10.1016/j.semarthrit.2010.08.005 21093020

[17] Dixon W, Watson K, Lunt M, Mercer L, Hyrich K, Symmons D (2010). Influence of anti-tumor necrosis factor therapy on cancer incidence in patients with rheumatoid arthritis who have had a prior malignancy: results from the British Society for Rheumatology Biologics Register. Arthritis Care Res.

[18] Strangfeld A, Hierse F, Rau R, Burmester G, Krummel-Lorenz B, Demary W, et al. Risk of incident or recurrent malignancies among patients with rheumatoid arthritis exposed to biologic therapy in the German biologics register RABBIT. Arthritis Res Ther. 2010;12:R5. 10.1186/ar2904 PMC2875631 20064207

[19] Wolfe F, Michaud K (2007). Biologic treatment of rheumatoid arthritis and the risk of malignancy: analyses from a large US observational study. Arthritis Rheum.

[20] Dreyer L, Mellemkjaer L, Andersen A, Bennett P, Poulsen U, Juulsgaard Ellingsen T, et al. Incidences of overall and site specific cancers in TNFalpha inhibitor treated patients with rheumatoid arthritis and other arthritides - a follow-up study from the DANBIO Registry. Ann Rheum Dis. 2013;72:79–82. 10.1136/annrheumdis-2012-201969 22945500

[21] Mariette X, Matucci-Cerinic M, Pavelka K, Taylor P, van Vollenhoven R, Heatley R, et al. Malignancies associated with tumour necrosis factor inhibitors in registries and prospective observational studies: a systematic review and meta-analysis. Ann Rheum Dis. 2011;70:1895–904. 10.1136/ard.2010.149419 21885875

[22] Ramiro S, Gaujoux-Viala C, Nam J, Smolen J, Buch M, Gossec L, et al. Safety of synthetic and biological DMARDs: A systematic literature review informing the 2013 update of the EULAR recommendations for management of rheumatoid arthritis. Ann Rheum Dis. 2014;73:529–35. 10.1136/annrheumdis-2013-204575 24401994

[23] Raaschou P, Simard J, Holmqvist M, Askling J, for the ARTIS Study Group (2013). Rheumatoid arthritis, anti-tumour necrosis factor therapy, and risk of malignant melanoma: nationwide population based prospective cohort study from Sweden. Br Med J.

[24] Lebrec H, Ponce R, Preston B, Iles J, Born T, Hooper M (2015). Tumor necrosis factor, tumor necrosis factor inhibition, and cancer risk. Curr Med Res Opin.

[25] Curado M, Edwards B, Shin H, Storm H, Ferlay J, Heanue M, et al. Cancer Incidence in Five Continents Vol. IX. Lyon, France: International Agengy for Research on Cancer; 2007.

[26] Buchbinder R, Barber M, Heuzenroeder L, Wluka A, Giles G, Hall S, et al. Incidence of melanoma and other malignancies among rheumatoid arthritis patients treated with methotrexate. Arthritis Rheum. 2008;59:794–9. 10.1002/art.23716 18512713

[27] Buchbinder R, March L, Lassere M, Briggs AM, Portek I, Reid C, Meehan A, Henderson L, Wengier L, van den Haak R. Effect of treatment with biological agents for arthritis in Australia: The Australian Rheumatology Association Database (ARAD). Int Med J. 2007;37:591-600. 10.1111/j.1445-5994.2007.01431.x 17573817

[28] Williams M, Buchbinder R, March L, Lassere M (2011). The Australian Rheumatology Association Database (ARAD). Sem Arthritis Rheum.

[29] Briggs A, March L, Lassere M, Reid C, Henderson L, Murphy B, et al. Baseline comorbidities in a population-based cohort of rheumatoid arthritis patients receiving biological therapy: data from the Australian rheumatology association database. Int J Rheumatol. 2009. doi: 10.1155/2009/861481. 10.1155/2009/861481 PMC2814100 20130803

[30] Staples M, March L, Lassere M, Reid C, Buchbinder R (2011). Health related quality of life and continuation rate on first line biologic therapy among rheumatoid arthritis patients from the Australian Rheumatology Association Database (ARAD). Rheumatol.

[31] Hawthorne G, Richardson J, Osborne R (1999). The Assessment of Quality of Life (AQoL) Instrument: a psychometric measure of health related quality of life. Qual Life Res.

[32] Fries J, Spitz P, Young D (1982). The dimensions of health outcomes: the Health Assessment Questionnaire, Disability and Pain Scales. J Rheumatol.

[33] National Cancer Statistics Clearing House Protocol. Protocol: Australian Institute of Health and Welfare; 2010.

[34] StataCorp. Stata Statistical Software: Release 11. College Station, TX: StataCorp LP; 2009.

[35] Gadalla S, Amr S, Langenberg P, Baumgarten M, Davidson W, Schairer C, et al. Breast cancer risk in elderly women with systemic autoimmune rheumatic diseases: a population-based case–control study. Br J Cancer. 2009;100:817–21. 10.1038/sj.bjc.6604906 PMC2651404 19190628

[36] Smitten A, Simon T, Hochberg M, Suissa S (2008). A meta-analysis of the incidence of malignancy in adult patients with rheumatoid arthritis. Arthritis Res Ther.

[37] Askling J, Fored CM, Brandt L, Baecklund E, Bertilsson L, Feltelius N, et al. Risks of solid cancers in patients with rheumatoid arthritis and after treatment with tumour necrosis factor antagonists. Ann Rheum Dis. 2005;64:1421–6. 10.1136/ard.2004.033993 PMC1755244 15829572

[38] Mercer L, Davies R, Galloway J, Low A, Lunt M, Dixon W, et al. Risk of cancer in patients receiving non-biologic disease-modifying therapy for rheumatoid arthritis compared with the UK general population. Rheumatol. 2013;51:91–8. 10.1093/rheumatology/kes350 PMC3521445 23238979

[39] Solomon D, Kremer J, Fisher M, Curtis J, Furer V, Harrold L, et al. Comparative cancer risk associated with methotrexate, other non-biologic and biologic disease-modifying anti-rheumatic drugs. Sem Arthritis Rheum. 2014;43:489–97. 10.1016/j.semarthrit.2013.08.003 24012043

[40] Mercer K, Lunt M, Low M, Dixon W, Watson K, Symmons D, Hyrich K, BSRBR Control Centre Consortium. Risk of solid cancer in patients exposed to anti-tumour necrosis factor therapy: results from the British Society for Rheumatology Biologics Register for Rheumatoid Arthritis. Ann Rheum Dis 2015;74:1087-93. 10.1136/annrheumdis-2013-204851 PMC4431340 24685910

[41] Madhusudan S, Foster M, Muthuramalingam S, Braybrooke J, Wilner S, Kaur K, et al. A phase II study of etanercept (Enbrel), a tumor necrosis factor alpha inhibitor in patients with metastatic breast cancer. Clin Cancer Res. 2004;10:6528–34. 10.1158/1078-0432.CCR-04-0730 15475440

[42] Perkins S, Cohen M, Rahme E, Bernatsky S. Melanoma and rheumatoid arthritis. Clin Rheumatol. 2012;31:1001–3. 10.1007/s10067-011-1908-x 22241727

[43] Rostom A, Dubé C, Lewin G, Tsertsvadze A, Barrowman N, Code C, et al. Nonsteroidal anti-inflammatory drugs and cyclooxygenase-2 inhibitors for primary prevention of colorectal cancer: a systematic review prepared for the U.S. Preventive Services Task Force. Ann Intern Med. 2007;146:376–89. 10.7326/0003-4819-146-5-200703060-00010 17339623

[44] Singh J, Furst D, Bharat A, Curtis J, Kavanaugh A, Kremer J, et al. 2012 update of the 2008 american college of rheumatology recommendations for the use of disease-modifying antirheumatic drugs and biologic agents in the treatment of rheumatoid arthritis. Arthritis Care Res. 2012;64:625–39. 10.1002/acr.21641 PMC4081542 22473917

